# Relationship between Hemoglobin Levels Corrected by Interdialytic Weight Gain and Mortality in Japanese Hemodialysis Patients: Miyazaki Dialysis Cohort Study

**DOI:** 10.1371/journal.pone.0169117

**Published:** 2017-01-03

**Authors:** Tatsunori Toida, Takashi Iwakiri, Yuji Sato, Hiroyuki Komatsu, Kazuo Kitamura, Shouichi Fujimoto

**Affiliations:** 1 Division of Circulatory and Body Fluid Regulation, Department of Internal Medicine, Faculty of Medicine, University of Miyazaki, Miyazaki, Japan; 2 Department of Hemovascular Medicine and Artificial Organs, Faculty of Medicine, University of Miyazaki, Miyazaki, Japan; 3 Department of Internal Medicine, Miyazaki Konan Hospital, Miyazaki, Japan; 4 Dialysis Division, University of Miyazaki Hospital, Miyazaki, Japan; 5 First Department of Internal Medicine, University of Miyazaki Hospital, Miyazaki, Japan; Tokushima University Graduate School, JAPAN

## Abstract

**Background:**

Although hemoglobin (Hb) levels are affected by a change in the body fluid status, the relationship between Hb levels and mortality while taking interdialytic weight gain (IDWG) at blood sampling into account has not yet been examined in hemodialysis patients.

**Study design:**

Cohort study.

**Setting, Participants:**

Data from the Miyazaki Dialysis cohort study, including 1375 prevalent hemodialysis patients (median age (interquartile range), 69 (60–77) years, 42.3% female).

**Predictor:**

Patients were divided into 5 categories according to baseline Hb levels and two groups based on the median value of IDWG rates at blood sampling at pre-HD on the first dialysis session of the week.

**Outcomes:**

All-cause and cardiovascular mortalities during a 3-year follow-up.

**Measurements:**

Hazard ratios were estimated using a Cox model for the relationship between Hb categories and mortality, and adjusted for potential confounders such as age, sex, dialysis duration, erythropoiesis-stimulating agent dosage, Kt/V, comorbid conditions, anti-hypertensive drug use, serum albumin, serum C-reactive protein, serum ferritin, and serum intact parathyroid hormone. Patients with Hb levels of 9–9.9 g/dL were set as our reference category.

**Results:**

A total of 246 patients (18%) died of all-cause mortality, including 112 cardiovascular deaths. Lower Hb levels (<9.0g/dL) were associated with all-cause mortality (adjusted HRs 2.043 [95% CI, 1.347–3.009]), while Hb levels were not associated with cardiovascular mortality. When patients were divided into two groups using the median value of IDWG rates (high IDWG, ≥5.4% and low IDWG, <5.4%), the correlation between lower Hb levels and all-cause mortality disappeared in high IDWG patients, but was maintained in low IDWG patients (adjusted HRs 3.058 [95% CI,1.575–5.934]). On the other hand, higher Hb levels (≥12g/dL) were associated with cardiovascular mortality in high IDWG patients (adjusted HRs 2.724 [95% CI, 1.010–7.349]), but not in low IDWG patients.

**Conclusion:**

In hemodialysis patients, target Hb levels may need to be selected in consideration of IDWG at blood sampling.

## Introduction

Anemia is common in end-stage renal disease and is a major risk factor that contributes to mortality in patients with chronic kidney disease. The optimal hemoglobin (Hb) target in these patients remains controversial. In many observational studies on hemodialysis (HD) patients, low Hb levels have been associated with mortality [1, 2], cardiovascular events [3], and quality of life [4, 5].

On the other hand, for higher Hb levels, an appropriate Hb target has remained under debate. Previous studies showed that higher Hb levels slightly increased the risk of death [6], and elevations in Hb levels have been implicated in a higher risk of mortality and cardiovascular events [7, 8]. Higher Hb targets have been suggested to reduce the need for transfusions, and have beneficial effects on quality of life [9–11]; however, disadvantages have also been reported [12–14].

Hb levels are known to vary during HD, and significantly differ when measured before or after dialysis or in the interdialysis period depending on ultrafiltration [15–18].

Although Hb levels are affected by a change in the body fluid status, the relationship between Hb levels and mortality while taking the interdialytic weight gain (IDWG) at blood sampling into account has not yet been examined. Therefore, the aim of the present study is to evaluate the relationships between Hb levels and all-cause and cardiovascular mortalities while adjusting for the effects of IDWG.

## Materials and Methods

**Fig 1:**
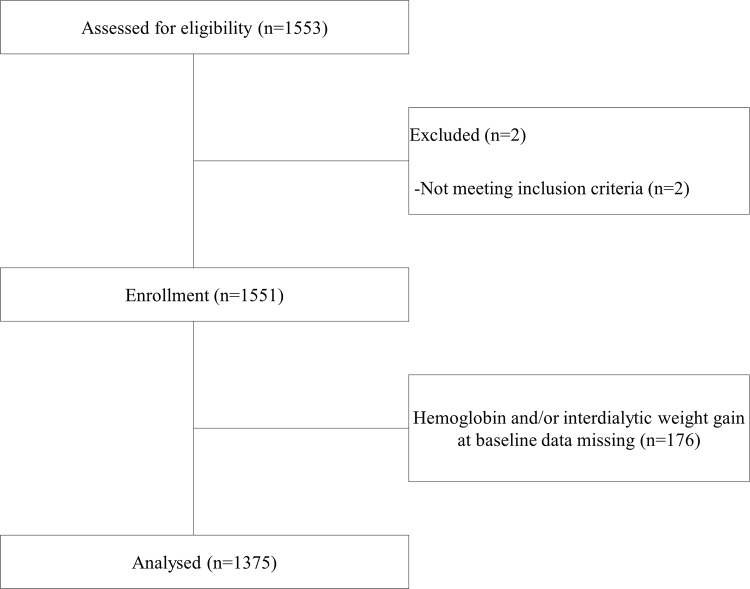
Flow of patients through the study.

The Miyazaki Dialysis Cohort study is a prospective observational study of maintenance HD patients from 27 dialysis centers and was initiated by the University of Miyazaki, Japan. A total of 1,375 patients were analyzed in this cohort study in December, 2009 and were followed-up for 3 years. Exclusion criteria included patients with a 3-month hemodialysis vintage, < 18 years of age, pregnant women, hospitalized patients, and patients not wishing to participate; 176 patients were excluded for missing Hb or IDWG data. Information on physical characteristics, laboratory data, basal renal diseases, comorbidities, and medications was collected by doctors in each dialysis center at the start of the study (Fig 1).

All causes of death were checked monthly by nursing staff or medical doctors during the follow-up period using questionnaires, which were searched by Y.S. and T.T. if necessary. Check sheets were collected annually. The survival time was defined as the time from enrollment to individual outcomes, the data for which were collected longitudinally during the course of the study follow-up until December 2012.

Cardiovascular mortality was defined as death from ischemic or hemorrhagic stroke, acute MI, causes related to congestive heart failure, sudden death, or aortic aneurysm rupture. Stroke was diagnosed using typical imaging and physical findings from examinations. Acute MI was diagnosed using typical electrocardiogram findings or elevations in myocardium-derived enzymes. Cardiac disease was confirmed based on a history of ischemic heart disease and/or congestive heart failure. Ischemic heart disease was defined as prior hospitalization or medication for angina pectoris and/or MI. Congestive heart failure was confirmed using electrocardiography, chest radiography, or echocardiography together with symptoms of dyspnea or edema. Sudden death was judged as unexpected death in the first hour following the start of symptoms or when the patient was found dead and had been seen alive 24 hours earlier. Blood samples are taken in a supine position at pre-HD on the first dialysis session of the week.

We converted the darbepoetin alfa dose to an equivalent epoetin alfa dose using a dose conversion ratio of 200 units of epoetin alfa per 1 μg of darbepoetin alfa [19, 20], and the doses of epoetin alfa and beta were considered to be equivalent.

Pre-HD blood pressure was measured in the supine position. Blood pressure values were averaged from 3 consecutive HD sessions during the week of patient enrollment.

### Statistical analysis

Descriptive analyses were calculated to describe variables such as patient characteristics in groups distributed according to Hb levels. All continuous variables were tested for a normal distribution, and the Student’s *t*-test (for a normal distribution) or Kruskal-Wallis test (for a non-normal distribution) or χ^2^ test was applied for comparisons of the five groups. Crude survival in a group was assessed using a Kaplan-Meier analysis with the Log-rank test. For Cox regression, our model included adjustment for age, sex, time on dialysis therapy, history of CVD, the presence of diabetes, serum albumin level. Given that previous studies found another covariates to be potential confounding factors, we also examined a model including these factors with anti-hypertension drugs, single-pool Kt/V, serum intact parathyroid hormone (iPTH), C-reactive protein and ferritin level [21–27]. All covariates were divided into categorical groups. We defined age as per 10 years, serum C-reactive protein, albumin, iPTH, ferritin, ESA dosage, time on dialysis therapy as quartile category. Patients with an Hb level of 9–9.9 g/dL were set as our reference category according to clinical guidelines [28]. All the covariates conformed to the proportional hazards model, using the Kaplan-Meier method and log–log plot. A multiple imputation approach using chained equations was used to account for missing covariates. All statistical analyses were performed with SPSS Statistics 20 (IBM Company, Chicago, USA).

### Ethical Considerations

This study was conducted in accordance with the principles contained in the Declaration of Helsinki and was approved by the University of Miyazaki Research Ethics Committee (No.516). Data collection was performed in a manner that maintained patient anonymity (UMIN00000516).

## Results

### Study participants and baseline characteristics

Table 1 shows baseline patient characteristics according to Hb levels. Significant differences were observed in age, sex, ESA use ratios, and the median values for serum albumin, creatinine, C-reactive protein, ferritin, i-PTH, single-pool Kt/V, and ESA dosage by the Kruskal-Wallis test or Student’s *t*-test. Seventeen patients out of 137 with an Hb level > 12 g/dl did not use ESA. No significant differences were observed in anti-hypertension drug use including angiotensin-converting enzyme inhibitors and angiotensin II receptor blockers.

**Table 1:** Baseline patient characteristics.

			Hemoglobin levels					p value*
	Number missing	Overall	<9 g/dL	9–9.9 g/dL	10–10.9 g/dL	11–11.9 g/dL	≥12 g/dL	
Number		1375	111	319	435	373	137	
Age (yr)	0	69 (60–77)	75 (68–82)	66 (60–74)	68 (58–76)	65 (55–74)	65 (55–74)	0.003
Female, n (%)	0	582 (42.3)	59 (53.2)	156 (48.9)	187 (43.0)	143 (38.3)	37 (27.0)	<0.001
Duration of HD (month)	17	75 (33–143)	66 (29–128)	72 (30–138)	79 (36–144)	77 (34–149)	74 (29–146)	0.642
Diabetes, n (%)	0	445 (32.4)	37 (33.3)	109 (34.2)	139 (32.0)	117 (31.4)	43 (31.4)	0.940
Pre-HD SBP (mmHg)	63	155.3 (143.0–167.3)	156.7 (145.0–166.3)	156.7 (143.7–170.0)	155.3 (144.0–166.0)	154.3 (142.0–168.0)	154.0 (136.3–164.2)	0.150
Previous history of CVD, n (%)	0	377 (27.4)	36 (32.4)	100 (31.3)	112 (25.7)	95 (25.5)	34 (24.8)	0.226
Hemoglobin (g/dL)	0	10.6 (9.7–11.4)	8.5 (7.9–8.7)	9.6 (9.3–9.8)	10.5 (10.2–10.7)	11.4 (11.2–11.6)	12.3 (12.1–12.8)	<0.001
Serum albumin (g/dL)	191	3.8 (3.6–4.0)	3.6 (3.4–3.9)	3.7 (3.5–4.0)	3.8 (3.6–4.1)	3.9 (3.6–4.1)	3.9 (3.6–4.1)	<0.001
Serum blood urine nitrogen (mg/dL)	24	65.8 (56.0–77.5)	64.2 (50.6–76.2)	65.3 (55.6–78.5)	64.3 (55.3–75.9)	68.2 (57.8–79.2)	66.3 (56.9–76.4)	0.067
Serum creatinine (mg/dL)	24	10.6 (8.8–12.5)	9.9 (7.9–11.4)	10.1 (8.6–11.6)	10.7 (9.0–12.4)	11.1 (9.3–13.1)	11.3 (9.1–13.7)	<0.001
Serum C-reactive protein (mg/dL)	286	0.20 (0.07–0.70)	0.40 (0.10–1.48)	0.31 (0.10–0.95)	0.19 (0.07–0.54)	0.13 (0.06–0.50)	0.20 (0.06–0.67)	<0.001
Transferrin saturation (%)	682	27.1 (18.6–37.2)	25.4 (14.1–36.7)	27.8 (19.5–38.4)	27.6 (19.0–37.8)	26.0 (18.3–36.1)	26.5 (17.9–35.6)	0.331
Serum Ferritin (ng/ml)	451	120.0 (44.5–257.0)	219.0 (91.8–404.5)	193.0 (55.3–332.6)	114.1 (45.4–227.8)	96.0 (39.1–212.0)	68.6 (34.3–158.8)	<0.001
Serum iPTH	556	150.8 (67.0–269.0)	136.0 (50.0–246.0)	144.0 (47.0–264.0)	148.0 (72.0–272.0)	149.0 (69.0–262.5)	190.6 (69.0–262.5)	0.033
Single-pool Kt/V	127	1.16 (1.03–1.32)	1.17 (1.00–1.33)	1.17 (1.03–1.32)	1.18 (1.03–1.33)	1.16 (1.04–1.31)	1.10 (0.96–1.26)	0.005
ESA use, n (%)	0	1299 (94.5)	110 (99.1)	308 (96.6)	411 (94.5)	350 (93.8)	120 (87.6)	0.001
ESA dosage (U/week)	542	3778 (2187–5705)	6000 (4000–8000)	4500 (3000–6000)	3224 (2250–5102)	3000 (2000–4486)	2925 (1500–4500)	<0.001
Anti-hypertensive drug use, n (%)	0	1183 (86.0)	88 (79.3)	277 (86.8)	369 (84.8)	321 (86.1)	118 (86.1)	0.388
-ACEI use, n (%)	0	124 (10.7)	8 (8.3)	30 (10.8)	32 (8.9)	45 (14.0)	9 (8.3)	0.191
-ARB use, n (%)	0	648 (54.2)	46 (46.9)	155 (54.8)	201 (54.2)	188 (56.1)	58 (53.2)	0.617
Interdialysis weight gain (%)	0	5.4 (4.2–6.7)	5.7 (4.0–7.5)	5.5 (4.5–7.2)	5.4 (4.0–6.6)	5.7 (4.5–7.0)	5.1 (4.4–6.5)	0.051

Continuous variables are represented as a median with the interquartile range in parentheses. Abbreviations: HD—hemodialysis, SBP—systolic blood pressure, iPTH—intact parathyroid hormone, ESA—erythropoiesis-stimulating agent.

* by the Kruskal-Wallis test or χ2 test.

In the 3 years after 1 January 2010, 246 patients died of all-cause mortality, including 112 cardiovascular deaths, while 111 (8.0%) moved to other dialysis facilities, including 16 who underwent kidney transplantation.

### Analysis for all-cause and cardiovascular mortalities

**Fig 2:**
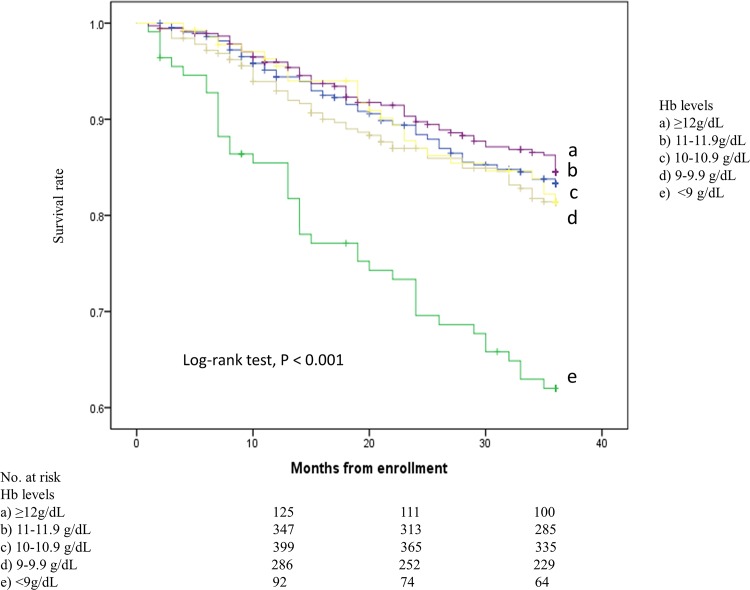
Kaplan–Meier estimates for survival rates among baseline Hb levels. The survival rate was significantly lower in patients in the Hb<9 g/dL group than in those in the other groups (Kaplan–Meier analysis, Log-rank test, P < 0.001).

Fig 2 shows that the survival rate was significantly lower in patients in the Hb < 9.0% group than in those in the other groups (Kaplan–Meier analysis, Log-rank test, P < 0.001). Unadjusted and adjusted Cox’s proportional hazard models showed that the risk of all-cause mortality was significantly higher in the groups with lower Hb levels (Hb<9.0%) (unadjusted HRs 2.366 [95% CI, 1.830–3.957], adjusted HRs 2.043 [95% CI, 1.347–3.099]) (Table 2A). S1 Table shows all HRs of covariates on all-cause mortality. On the other hand, unadjusted Cox’s proportional hazard models showed that the risk of cardiovascular mortality was significantly higher in the groups with lower Hb levels (Hb<9.0%) (HRs 1.960 [95% CI, 1.008–3.809]); however, cardiovascular mortality was not correlated with the Hb level after multivariate adjustment (Table 2B).

**Table 2:** Relationship between baseline hemoglobin levels and hazard ratios of all-cause (2a) and cardiovascular (2b) mortalities.

		Hemoglobin level				
		<9 g/dL	9–9.9 g/dL	10–10.9 g/dL	11–11.9 g/dL	≥12 g/dL
2a)	Unadjusted model	2.366 (1.830–3.957)**	1.000 (ref)	0.879 (0.619–1.249)	0.803 (0.553–1.164)	0.970 (0.601–1.565)
	Adjusted model*	2.043 (1.347–3.009)**	1.000 (ref)	1.109 (0.766–1.607)	1.226 (0.821–1.829)	1.374 (0.823–2.293)
2b)	Unadjusted model	1.960 (1.008–3.809)**	1.000 (ref)	1.164 (0.693–1.953)	0.961 (0.551–1.676)	0.985 (0.469–2.068)
	Adjusted model*	1.823 (0.920–3.609)	1.000 (ref)	1.417 (0.820–2.451)	1.394 (0.766–2.539)	1.266 (0.575–2.766)

Values shown are hazard ratios (95% confidence interval).

*Adjusted for age, sex, ESA dosage, time on dialysis therapy, diabetes, previous history of cardiovascular diseases, single pool Kt/V, serum Alb, serum ferritin, serum C-reactive protein, serum iPTH and anti-hypertensive drug.

** p value <0.05.

### Effects of IDWG at blood sampling on the relationship between Hb levels and mortality

Dividing the patients into two groups using the median value of IDWG rates (high IDWG, ≥5.4% and low IDWG, <5.4%), the all-cause mortality of high IDWG group was significantly higher compared with low IDWG group (Kaplan-Meier analysis, P<0.001). However, there was no significant correlation between Hb levels and IDWG (0.040 by Pearson's correlation coefficient, p = 0.135), and these factors have no interaction on all-cause and cardiovascular mortality (S1 Fig). Baseline patient characteristics according to Hb levels were shown in Table 3. In the low IDWG group, significant differences were observed in sex, serum albumin, serum creatinine, serum C-reactive protein, serum ferritin, single-pool Kt/V, ESA use, and ESA dosage. On the other hand, in the high IDWG group, significant differences were observed in sex, age, pre-HD systolic blood pressure, serum albumin, serum blood urine nitrogen, serum creatinine, serum C-reactive protein, serum ferritin, ESA use, ESA dosage, and IDWG.

**Table 3:** Baseline patient characteristics stratified by the interdialytic weight gain status.

	IDWG<5.4%						IDWG>5.4%					
	<9 g/dL	9–9.9 g/dL	10–10.9 g/dL	11–11.9 g/dL	≥12 g/dL	p value*	<9 g/dL	9–9.9 g/dL	10–10.9 g/dL	11–11.9 g/dL	≥12 g/dL	p value*
Number	51	153	217	170	82		60	166	218	203	55	
Age (yr)	74 (62–80)	69 (62–78)	68 (59–76)	66 (59–76)	70 (60–78)	0.060	75 (65–78)	69 (61–78)	69 (58–77)	67 (59–76)	66 (55–74)	0.026
Female, n (%)	26 (51.0)	73 (47.7)	91 (41.9)	62 (36.5)	19 (23.2)	0.002	33 (55.0)	83 (50.0)	96 (44.0)	81 (39.9)	18 (32.7)	0.049
Duration of HD (month)	68 (29–138)	61 (27–134)	62 (32–134)	77 (31–152)	55 (20–138)	0.753	62 (29–128)	81 (36–148)	93 (44–151)	81 (35–142)	88 (38–165)	0.182
Diabetes, n (%)	13 (25.5)	50 (32.7)	69 (31.8)	47 (27.6)	24 (29.3)	0.771	24 (40.0)	59 (35.5)	70 (32.1)	70 (34.5)	19 (34.5)	0.838
Pre-HD SBP (mmHg)	155.3 (143.3–166.3)	152.0 (141.7–164.5)	154.7 (144.5–164.0)	152.7 (140.0–163.1)	154.0 (135.8–164.0)	0.679	157.0 (143.9–166.2)	160.6 (150.0–173.8)	156.2 (144.4–168.0)	155.3 (145.7–171.3)	154.0 (136.7–164.7)	0.019
Previous history of CVD, n (%)	18 (35.3)	51 (33.3)	53 (24.4)	38 (22.4)	23 (28.0)	0.112	18 (30.0)	49 (29.5)	59 (27.1)	57 (28.1)	11 (20.0)	0.714
Hemoglobin (g/dL)	8.5 (7.8–8.7)	9.6 (9.3–9.8)	10.5 (10.2–10.7)	11.4 (11.2–11.6)	12.3 (12.1–12.7)	<0.001	8.5 (8.0–8.8)	9.5 (9.3–9.7)	10.5 (10.2–10.7)	11.4 (11.2–11.7)	12.4 (12.1–12.9)	<0.001
Serum albumin (g/dL)	3.6 (3.4–4.0)	3.8 (3.5–4.0)	3.8 (3.6–4.1)	3.9 (3.7–4.1)	3.9 (3.6–4.1)	0.001	3.6 (3.3–3.8)	3.7 (3.5–4.0)	3.8 (3.6–4.0)	3.9 (3.6–4.1)	3.9 (3.6–4.1)	<0.001
Serum blood urine nitrogen (mg/dL)	64.9 (49.2–77.5)	62.4 (50.8–73.1)	62.7 (53.0–73.4)	66.0 (53.1–78.1)	64.4 (52.0–72.9)	0.567	62.3 (53.1–75.1)	69.4 (60.1–83.0)	66.6 (57.5–78.1)	69.4 (61.2–81.6)	69.8 (61.6–82.3)	0.009
Serum creatinine (mg/dL)	10.1 (7.9–11.8)	10.1 (8.7–11.5)	10.6 (9.0–12.2)	11.2 (9.1–13.5)	10.9 (8.9–13.3)	0.002	9.6 (7.9–11.1)	10.2 (8.5–11.6)	10.8 (9.0–12.6)	10.9 (9.3–12.8)	12.5 (9.2–14.4)	<0.001
Transferrin saturation (%)	27.1 (12.3–34.9)	28.5 (21.0–38.8)	27.4 (19.9–35.1)	26.7 (17.8–38.0)	25.8(16.8–34.3)	0.427	25.1 (16.3–37.8)	26.0 (18.5–38.5)	28.7 (18.9–40.3)	25.7 (18.5–34.5)	27.0 (18.0–37.6)	0.435
Serum C-reactive protein (mg/dL)	0.52 (0.20–1.40)	0.40 (0.10–1.39)	0.17 (0.06–0.70)	0.15 (0.06–0.70)	0.20 (0.08–1.15)	<0.001	0.30 (0.10–0.98)	0.21 (0.10–0.81)	0.20 (0.07–0.56)	0.10 (0.05–0.45)	0.10 (0.04–0.40)	0.003
Serum ferritin (ng/ml)	219.0 (70.1–378.6)	210.6 (59.0–321.7)	112.6 (45.9–218.8)	92.2 (39.4–218.1)	73.9 (37.4–148.8)	<0.001	227.0 (108.8–405.6)	176.3 (51.5–344.4)	116.3 (45.3–256.9)	98.2 (38.9–201.2)	66.7 (27.1–170.8)	<0.001
Serum iPTH (pg/ml)	129.0 (45.9–246.0)	144.0 (51.0–284.0)	147.0 (72.5–256.6)	161.5 (67.6–287.0)	192.3 (110.0–312.3)	0.085	143.0 (60.0–250.8)	148.0 (45.2–261.1)	150.1 (70.1–292.7)	142.4 (70.0–235.0)	188.0 (102.0–293.2)	0.344
Single-pool Kt/V	1.21 (1.03–1.33)	1.13 (1.02–1.30)	1.16 (1.02–1.32)	1.16 (1.01–1.27)	1.10 (0.95–1.25)	0.043	1.12 (1.00–1.34)	1.19 (1.04–1.32)	1.20 (1.06–1.35)	1.17 (1.04–1.35)	1.11 (0.97–1.26)	0.116
ESA use, n (%)	51 (100)	147 (96.1)	209 (96.3)	157 (92.4)	72 (87.8)	0.008	59 (98.3)	161 (97.0)	202 (92.7)	193 (95.1)	48 (87.3)	0.031
ESA dosage (U/week)	6000 (3750–8000)	5074 (3000–6872)	3000 (2599–4754)	3000 (1603–4027)	2909 (1559–4500)	<0.001	6000 (4500–8158)	4182 (3000–6000)	3251 (2000–5455)	3000 (2000–4500)	3000 (1339–5027)	<0.001
Anti-hypertensive drug use, n (%)	39 (76.5)	131 (85.6)	190 (87.6)	145 (85.3)	69 (84.1)	0.386	49 (81.7)	146 (88.0)	179 (82.1)	176 (86.7)	49 (89.1)	0.369
Interdialysis weight gain (%)	3.7 (2.4–4.8)	4.4 (3.5–4.9)	4.2 (3.3–4.8)	4.2 (3.5–4.9)	4.4 (3.5–4.9)	0.161	6.9 (6.1–8.5)	7.0 (6.1–7.9)	6.6 (5.9–7.4)	6.6 (5.9–7.9)	6.9 (5.8–7.5)	0.028

Continuous variables are represented as a median with the interquartile range in parentheses. Abbreviations: HD—hemodialysis, SBP—systolic blood pressure, CVD–cardiovascular disease, iPTH—intact parathyroid hormone, ESA—erythropoiesis-stimulating agent.

* by the Kruskal-Wallis test or χ2 test.

Table 4 shows the numbers of all-cause and cardiovascular deaths. The correlation between lower Hb levels and all-cause mortality disappeared in high IDWG patients (IDWG ≥5.4%), but was maintained in low IDWG patients (<5.4%) (adjusted HRs 3.058 [95% CI, 1.575–5.934]) (Table 5A). On the other hand, higher Hb levels (≥12g/dL) were associated with cardiovascular mortality in high IDWG patients (adjusted HRs 2.365 [95% CI, 0.087–9.9820.882–6.344]), but not in low IDWG group. (Table 5B). In higher Hb group among high IDWG group, serum ferritin levels at baseline were significantly different between the patients with or without cardiovascular death (with cardiovascular death vs. without: median (interquartile range) 232.7 (63.1–383.9) vs 56.8 (26.6–130.9), p = 0.022, Wilcoxon signed-rank test), but other covariates were not different (S2 Table).

**Table 4:** Number of all-cause and cardiovascular deaths and observed Hb levels stratified by the interdialytic weight gain (IDWG) status.

	IDWG<5.4%					IDWG≥5.4%				
	<9 g/dL (n = 51)	9–9.9 g/dL (n = 153)	10–10.9 g/dL (n = 217)	11–11.9 g/dL (n = 170)	≥12 g/dL (n = 82)	<9 g/dL (n = 60)	9–9.9 g/dL (n = 166)	10–10.9 g/dL (n = 218)	11–11.9 g/dL (n = 203)	≥12 g/dL (n = 55)
All-cause death, n (%)	20 (39.2)	21 (13.7)	29 (13.4)	21 (12.4)	13 (15.6)	21 (35.0)	35 (21.1)	41 (18.8)	34 (16.7)	11 (20.0)
Cardiovascular death, n (%)	9 (17.6)	11 (7.2)	18 (8.3)	12 (7.1)	3 (3.7)	5 (8.3)	12 (7.2)	20 (9.2)	15 (7.4)	7 (12.7)

**Table 5:** Relationship between baseline hemoglobin levels and adjusted hazard ratios of all-cause (5a) and cardiovascular (5b) mortalities by the category of the interdialytic weight gain status.

		Hemoglobin level				
		<9 g/dL	9–9.9 g/dL	10–10.9 g/dL	11–11.9 g/dL	≥12 g/dL
5a)	IDWG<5.4%	3.058 (1.575–5.934)*	1.000 (ref)	1.156 (0.623–2.143)	1.504 (0.767–2.949)	1.374 (0.622–3.003)
	IDWG≥5.4%	1.367 (0.760–2.461)	1.000 (ref)	1.061 (0.661–1.702)	1.061 (0.640–1.758)	1.511 (0.744–3.067)
5b)	IDWG<5.4%	2.359 (0.895–6.221)	1.000 (ref)	1.025 (0.450–2.336)	1.349 (0.543–3.353)	0.395 (0.100–1.555)
	IDWG≥5.4%	1.061 (0.355–3.171)	1.000 (ref)	1.570 (0.744–3.314)	1.439 (0.640–3.235)	2.724 (1.010–7.349)*

Values shown are hazard ratios (95% confidence interval). Adjusted for age, sex, ESA dosage, time on dialysis therapy, diabetes, previous history of cardiovascular disease, single pool Kt/V, serum Alb, serum ferritin, serum C-reactive protein, serum iPTH and anti-hypertensive drugs.

*p value <0.05.

## Discussion

In the present prospective cohort study on HD patients, lower Hb levels (<9.0 g/dL) were associated with all-cause mortality. This relationship remained largely unchanged even after adjustments for the potential confounding factors of age, sex, dialysis duration, ESA dosage, Kt/V, comorbid conditions, anti-hypertensive drug use, serum albumin, C-reactive protein, ferritin and iPTH. When patients were divided into two groups using the median value of IDWG rates (high IDWG, ≥5.4% and low IDWG, <5.4%), the correlation between lower Hb levels and all-cause mortality disappeared in high IDWG patients, but was maintained in low IDWG patients. On the other hand, higher Hb levels were associated with cardiovascular mortality in high IDWG patients, but not in low IDWG patients.

In an international comparison of Hb levels and ESA use in HD patients, despite major differences in the treatments used [29,30], several observational studies showed that severe anemia in HD patients was related to increased morbidity and mortality [31–35]. In Japan and other Asian countries, several observational studies reported similar findings [36–39]. Although the relationship between lower Hb levels and the risk of mortality is well known in HD patients, an appropriate Hb target as higher Hb levels has remained controversial in terms of cardiovascular events in addition to mortality. Three large randomized controlled trials using patients with chronic kidney disease not yet on dialysis have been completed [40–42] and found no evidence of benefits of a higher (compared with lower) Hb target on cardiovascular events or a composite outcome of death, myocardial infarction, or hospitalization for congestive heart failure and stroke, or found an increased risk of adverse events. In HD patients, the Normal Hematocrit Trial [8], a study in which more than 1200 patients with congestive heart failure or ischemic heart disease were randomized to target Hct values of 42% (normal Hct group) and 30%, had to be stopped prematurely by the Data Safety Monitoring Board because of concerns regarding increased risks of cardiovascular disease and mortality in the normal Hct arm. On the other hand, in HD patients with naturally occurring higher Hb levels, Goodkin et al. reported that Hb levels of 12 g/dl did not increase the risk of mortality in patients without ESA therapy over that in other patients [43].

ESA-sensitive patients derive survival benefits from the full correction of anemia, whereas patients with ESA resistance (which is a predictor of poor survival) may be harmed by the high doses of ESA prescribed in the attempt to increase Hb levels [44, 45]; however, this is still under debate [46]. Although higher Hb targets have been suggested to reduce the need for transfusions and have beneficial effects on the quality of life of patients [9–11], disadvantages have also been reported [12–14]. In addition to the use of ESA, several studies have shown that the survival benefit is affected by age [47], diabetes [48] and a previous history of cardiovascular disease [49].

A failure in the management of body fluid volume has a negative impact on blood pressure and the cardiovascular system [50–53]. Furthermore, there are some reports that analyzed the association between IDWG and prognosis. According to United States Renal Data System data, high IDWG (>4.8%) is associated with mortality [54]. In other studies, IDWG of more than 5.7% has been associated with a higher risk of adverse outcomes [55]. In the present study, higher IDWG (≥5.4%) was associated with all-cause mortality (Kaplan-Meier analysis, P<0.001).

Serum Hb levels have been shown to vary during HD, and Hb levels also significantly differ when measured before or after dialysis or in the interdialysis period [15–18]. Vlassopoulos et al. [15] measured Hb levels in 15 stable patients before the initiation of a HD session, and at 24, 48, and 72 hours in the interdialysis period. They observed a significant 24-h postdialysis increase in Hb levels from the predialysis level, with a gradual decrease to a non-significant level before the next HD session. Movilli et al. reported similar findings [16]. Bellizzi et al. demonstrated that interdialytic Hb increments differed in the three sessions and weight loss per 1L of ultrafiltration led to an increase in the Hb level of approximately 0.4 g/dL [17]. Castillo also reported a significant increment in Hb levels after HD. The mean percent increase in Hb levels was 6.1%, and increased to approximately 9% in patients with a body weight loss ≥ 2.5 kg/session [18]. Considering above-mentioned reports, the present study that demonstrated a correlation between cardiovascular mortality and higher Hb levels in high IDWG patients may suggest that an over-correction of Hb after HD is correlated to cardiovascular death. Higher Hb levels after HD could be reached resulting in severe hypertension and hypercoagulability or decreased perfusion to major arteries already at risk [56–58], and then be related to cardiovascular events. However, either high IDWG or high Hb levels of pre-HD are more involved in the higher Hb levels of post-HD is unknown in this study, and the definite relationship between cardiovascular death and the Hb level of post-HD remains unclear. Further studies will be needed to clarify the relationships between the post-HD Hb levels and cardiovascular events. In higher Hb group among high IDWG group, serum ferritin levels were higher in the patients with cardiovascular death than without. Recent study showed that the patients with higher serum ferritin levels were associated with higher mortality risk than those with lower ferritin levels [59]. Therefore, high serum ferritin levels may accelerate cardiovascular mortality in higher Hb group among high IDWG group.

The present study is the first prospective study to have examined the relationship between mortality and Hb levels in consideration of IDWG at blood sampling. Furthermore, multivariate analyses on mortality were performed after adjustments for some known confounding factors of all-cause and cardiovascular mortalities other than Hb levels (age, sex, dialysis duration, diabetes, Kt/V, comorbid conditions, ACEI/ARB use, serum albumin, C-reactive protein, ferritin, i-PTH, and ESA dosage) [21–27].

This study has several limitations. The present study was observational, not interventional. Furthermore, the result of this study may be limited only to the registration facility. It is necessary to examine external validity. Our cohort consisted of prevalent, but not incident chronic dialysis patients. Therefore, some patients with severe anemia control may have died before study enrollment. Moreover, a major cause of cardiovascular mortality is congestive heart failure; however, we were unable to identify the specific underlying etiologies. Thus, there may have been some valvular heart diseases in addition to ischemic heart diseases; it is unclear how Hb levels affect valvular heart diseases. Another limitation is that baseline data were used to define exposure categories in this cohort, and we were unable to examine the effects of changes from the baseline category during follow-up, although the average value of Hb and IDWG may be appropriate than single measurement. Equation between epoetin and darbepoetin [19, 20] is not established one.

## Conclusions

The results of the present study demonstrated that lower Hb levels (<9.0g/dL) were associated with all-cause mortality in low IDWG patients only. Furthermore, regarding cardiovascular mortality, a correlation was observed in lower Hb levels in low IDWG patients and higher Hb levels in high IDWG patients. Although Hb levels are affected by a change in the body fluid status, the target Hb of the current guidelines [27, 60, 61] do not take into account body fluid status. The results of the present study suggest that target Hb levels may need to be selected in consideration of IDWG at blood sampling.

## Supporting Information

S1 Fig Interaction of Hb and IDWG on all-cause mortality (a) and cardiovascular mortality (b).
(TIF)

S1 Table Relationship between covariates and hazard ratios of all-cause mortality. (DOCX)

S2 Table Comparison of the patients characteristics between the patients with cardiovascular deaths in higher Hb group among high IDWG group. (DOCX)
