# Supplementary figures and images for: Relationship between Hemoglobin Levels Corrected by Interdialytic Weight Gain and Mortality in Japanese Hemodialysis Patients: Miyazaki Dialysis Cohort Study
Source: PLoS One. 2017 Jan 3;12(1):e0169117. doi: 10.1371/journal.pone.0169117 (PMC5207402; doi:10.1371/journal.pone.0169117)

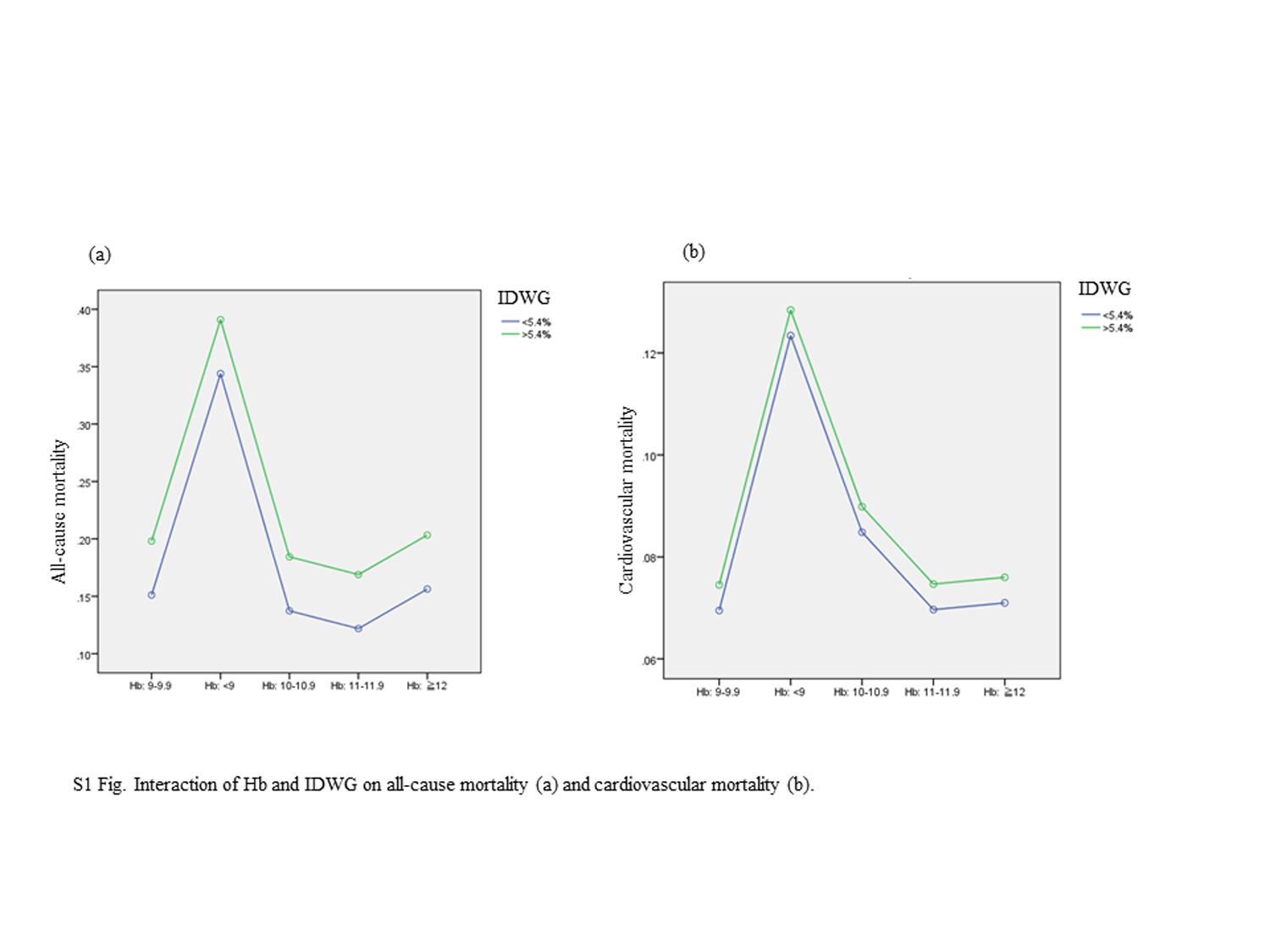

Supplement: S1 Fig — Interaction of Hb and IDWG on all-cause mortality (a) and cardiovascular mortality (b). (TIF) [file pone.0169117.s001.tif]
