# Supplementary material for: Relationship between Hemoglobin Levels Corrected by Interdialytic Weight Gain and Mortality in Japanese Hemodialysis Patients: Miyazaki Dialysis Cohort Study
Source: PLoS One. 2017 Jan 3;12(1):e0169117. doi: 10.1371/journal.pone.0169117 (PMC5207402; doi:10.1371/journal.pone.0169117)
Supplement: S1 Table — (DOCX) [file pone.0169117.s002.docx]

| Covariates | HRs | 95%CI |
| --- | --- | --- |
| male | 0.915 | 0.678-1.234 |
| age (per 10 y) | 1.662 | 1.455-1.898 |
| diabetes | 1.314 | 0.992-1.741 |
| Duration of HD (momths) |  |  |
| Q1 (<33) | 1.000 (ref) |  |
| Q2 (33-75) | 1.144 | 0.816-1.602 |
| Q3 (76-143) | 0.861 | 0.591-1.254 |
| Q4 (>143) | 0.930 | 0.616-1.405 |
| History of CVD | 1.353 | 1.033-1.898 |
| Anti-hypertension drug | 0.979 | 0.682-1.407 |
| ESA dose (U/week) |  |  |
| Q1 (<2206) | 1.000 (ref) |  |
| Q2 (2206-3787) | 1.524 | 0.996-2.331 |
| Q3 (3787-6000) | 1.292 | 0.854-1.954 |
| Q4 (>6000) | 1.952 | 1.287-2.960 |
| Single-pool Kt/V |  |  |
| Q1 (<1.03) | 1.000 (ref) |  |
| Q2 (1.03-1.16) | 1.048 | 0.729-1.505 |
| Q3 (1.16-1.32) | 1.098 | 0.753-1.600 |
| Q4 (≥1.32) | 1.952 | 0.572-1.396 |
| Albumin (g/dL) |  |  |
| Q1 (<3.61) | 1.000 (ref) |  |
| Q2 (3.61-3.80) | 0.595 | 0.417-0.849 |
| Q3 (3.80-4.00) | 0.596 | 0.425-0.836 |
| Q4 (≥4.00) | 0.564 | 0.368-0.865 |
| C-reactive protein (mg/dL) |  |  |
| Q1 (<0.07) | 1.000 (ref) |  |
| Q2 (0.08-0.20) | 1.614 | 1.082-2.433 |
| Q3 (0.20-0.64) | 1.988 | 1.120-2.627 |
| Q4 (≥0.64) | 2.191 | 1.296-2.970 |
| Ferritin (ng/mL) |  |  |
| Q1 (<44.5) | 1.000 (ref) |  |
| Q2 (44.5-116.0) | 1.248 | 0.837-1.862 |
| Q3 (116.0-248.9) | 1.478 | 0.998-2.189 |
| Q4 (>248.9) | 1.098 | 0.741-1.627 |
| Intact parathyroid hormon |  |  |
| Q1 (<69) | 1.000 (ref) |  |
| Q2 (69-158) | 0.794 | 0.556-1.134 |
| Q3 (158-273) | 1.022 | 0.723-1.445 |
| Q4 (>273) | 1.018 | 0.706-1.468 |

S1 Table. Relationship between covariates and hazard ratios of all-cause mortality.

Abbreviations: HD - hemodialysis, CVD – cardiovascular disease, Q1 – first quartile, Q2 – second quartile, Q3 – third quartile, Q4 – fourth quartile, ESA - erythropoiesis-stimulating agent.
