# Supplementary material for: Relationship between Hemoglobin Levels Corrected by Interdialytic Weight Gain and Mortality in Japanese Hemodialysis Patients: Miyazaki Dialysis Cohort Study
Source: PLoS One. 2017 Jan 3;12(1):e0169117. doi: 10.1371/journal.pone.0169117 (PMC5207402; doi:10.1371/journal.pone.0169117)
Supplement: S2 Table — (DOCX) [file pone.0169117.s003.docx]

S2 Table. Comparison of the patients characteristics between the patients with cardiovascular deaths in higher Hb group among high IDWG group.

|  | Cardiovascular death | | p value* |
| --- | --- | --- | --- |
|  | (+) | (-) |  |
| Number | 7 | 48 |  |
| Age (yr) | 77.0 (66.0-85.0) | 64.0 (55.0-71.8) | 0.065 |
| Female, n (%) | 4 (57.1) | 14 (29.2) | 0.141 |
| Duration of HD (month) | 111 (42-228) | 88 (35-165) | 0.544 |
| Diabetes, n (%) | 4 (57.1) | 15 (31.2) | 0.178 |
| Pre-HD SBP (mmHg) | 150.0 (130.7-180.7) | 154.7 (136.8-164.6) | 0.649 |
| Previous history of CVD, n (%) | 1 (14.3) | 10 (20.8) | 0.686 |
| Hemoglobin (g/dL) | 12.4 (12.0-12.5) | 12.4 (12.2-13.1) | 0.233 |
| Serum albumin (g/dL) | 3.9 (3.8-4.1) | 3.9 (3.5-4.1) | 0.355 |
| Serum C-reactive protein (mg/dL) | 0.25 (0.03-1.22) | 0.10 (0.04-0.38) | 0.516 |
| Serum Ferritin (ng/mL) | 232.7 (63.1-383.9) | 56.8 (26.6-130.9) | 0.022 |
| Serum intact parathyroid hormone (pg/mL) | 245.4 (162.0-285.0) | 181.0 (93.3-295.9) | 0.419 |
| Serum total choresterol (mg/dL) | 162.0 (143.7-169.0) | 157.0 (138.2-177.7) | 0.791 |
| Serum LDL choresterol (mg/dL) | 78.0 (66.3-95.0) | 81.7 (65.8-94.9) | 0.990 |
| Serum triglyceride (mg/dL) | 84.0 (64.0-119.0) | 94.0 (54.8-131.5) | 0.960 |
| Single-pool Kt/V | 1.26 (1.16-1.31) | 1.09 (0.96-1.25) | 0.051 |
| ESA use, n (%) | 5 (71.4) | 43 (89.6) | 0.178 |
| ESA dosage (U/week) | 3750 (0-5427) | 2963 (1425-4438) | 0.960 |
| Anti-hypertensive drug use, n (%) | 6 (85.7) | 43 (89.6) | 0.759 |
| Interdialysis weight gain (%) | 7.1 (5.9-9.2) | 6.8 (5.9-7.5) | 0.752 |

Continuous variables are represented as a median with the interquartile range in parentheses.

* by the Kruskal-Wallis test or χ2 test.

Abbreviations: HD - hemodialysis, SBP - systolic blood pressure, CVD – cardiovascular disease, iPTH - intact parathyroid hormone, ESA - erythropoiesis-stimulating agent.
